# Adipose-derived stem cell exosome NFIC improves diabetic foot ulcers by regulating miR-204-3p/HIPK2

**DOI:** 10.1186/s13018-023-04165-x

**Published:** 2023-09-14

**Authors:** Huimin Huang, Wufei Zhu, Zongwei Huang, Dengze Zhao, Lu Cao, Xian Gao

**Affiliations:** 1 Burn, Plastic and Wound Surgery Department, Yichang Central People’s Hospital, The First College of Clinical Medical Science, China Three Gorges University, Yichang, China; 2 Department of Endocrinology, Yichang Central People’s Hospital, The First College of Clinical Medical Science, China Three Gorges University, Yichang, China; 3 Burn, Plastic and Wound Surgery Department, Huanggang Central Hospital of Yangtze University, No.126, Qian Avenue, Huangzhou District, Huanggang, 438000 Hubei China

**Keywords:** ADSC, NFIC, Diabetic foot ulcers, HUVEC, miR-204-3p, HIPK2

## Abstract

**Background:**

Diabetic foot ulcers (DFU) are a serious complication of diabetes that lead to significant morbidity and mortality. Recent studies reported that exosomes secreted by human adipose tissue-derived mesenchymal stem cells (ADSCs) might alleviate DFU development. However, the molecular mechanism of ADSCs-derived exosomes in DFU is far from being addressed.

**Methods:**

Human umbilical vein endothelial cells (HUVECs) were induced by high-glucose (HG), which were treated with exosomes derived from nuclear factor I/C (NFIC)-modified ADSCs. MicroRNA-204-3p (miR-204-3p), homeodomain-interacting protein kinase 2 (HIPK2), and NFIC were determined using real-time quantitative polymerase chain reaction. Cell proliferation, apoptosis, migration, and angiogenesis were assessed using cell counting kit-8, 5-ethynyl-2′-deoxyuridine (EdU), flow cytometry, wound healing, and tube formation assays. Binding between miR-204-3p and NFIC or HIPK2 was predicted using bioinformatics tools and validated using a dual-luciferase reporter assay. HIPK2, NFIC, CD81, and CD63 protein levels were measured using western blot. Exosomes were identified by a transmission electron microscope and nanoparticle tracking analysis.

**Results:**

miR-204-3p and NFIC were reduced, and HIPK2 was enhanced in DFU patients and HG-treated HUVECs. miR-204-3p overexpression might abolish HG-mediated HUVEC proliferation, apoptosis, migration, and angiogenesis in vitro. Furthermore, HIPK2 acted as a target of miR-204-3p. Meanwhile, NFIC was an upstream transcription factor that might bind to the miR-204-3p promoter and improve its expression. NFIC-exosome from ADSCs might regulate HG-triggered HUVEC injury through miR-204-3p-dependent inhibition of HIPK2.

**Conclusion:**

Exosomal NFIC silencing-loaded ADSC sheet modulates miR-204-3p/HIPK2 axis to suppress HG-induced HUVEC proliferation, migration, and angiogenesis, providing a stem cell-based treatment strategy for DFU.

## Introduction

As one of the most severe complications of diabetes, diabetic foot ulcers (DFU) belong to lower extremity vascular disease accompanied by a major risk of infection amputation, and death [1, 2]. At present, DFU is becoming a worldwide public health challenge, threatening 9.1–26.1 million individuals with diabetes annually [3], with the greatest prevalence in patients ages 45 and over. Clinically, it is characterized by skin lesions, gangrene, necrosis, and even after healing there is still a high recurrence rate and amputation [4]. Recently, some risk factors, containing poor blood sugar control, neuropathy, ischemia, trauma, and local infection, have been considered to be involved in the formation of this disease [5]. Despite remarkable advances in conventional treatments, including wound dressing, hyperbaric oxygen therapy, sensitive antibiotics usage, and wound debridement [6–8], which have relieved and delayed the progression of this disease, persistent non-healing foot ulcers frequently occur in these patients [9]. A recent study displayed that poor angiogenesis is directly associated with high glucose (HG) levels [10]. Furthermore, endothelial cell function is impaired due to increased inhibition of proliferation and migration and apoptosis triggered by HG [11]. Accordingly, promoting wound healing is regarded as a promising strategy for the prevention of DFU patients.

During the past decades, there has been increasing attention to the functional role of adipose tissue-derived mesenchymal stem cells (ADSCs), which are adult stem cells with high regenerative capacity identified in adipose tissues [12]. In addition, mesenchymal stem cell-derived extracellular vesicles might be involved in the management of inflammatory, autoimmune, and regenerative medicine [13, 14]. Due to their easy availability, limited replicative lifespan, and absence of ethical concerns, ADSCs have been previously published as beneficial for the treatment of various human diseases [15, 16]. Lately, it has become apparent that, in addition to partaking in immune modulation and reducing inflammation, ADSCs also might improve the process of wound healing via differentiating into fibroblasts and endothelial cells or via secreting growth factors to recruit endogenous cells and enhance fibroblasts proliferation [17]. Beyond that, it has been reported that ADSC-derived exosomes might be internalized by fibroblasts to induce cell migration, proliferation, and collagen synthesis, thereby accelerating cutaneous wound healing [18]. Notably, exosomes from ADSCs overexpressing Nrf2 (a transcription factor) had been validated to facilitate the healing of DFU via increasing vascularization in a rat model [19]. Hence, ADSCs-derived exosome therapy might be a promising novel method for treating DFU [20], but its exact efficacy is poorly defined.

As a much-explored type of naturally occurring non-coding RNAs, microRNAs (miRNAs) have emerged as powerful gene regulators in diverse cellular activities [21]. Canonically, miRNAs might trigger mRNA degradation and translational delaying via incomplete or complete base pairing to the 3′ UTR of mRNA [22]. Recent evidence has suggested that miRNAs play a vital role in musculoskeletal conditions [23–26]. Furthermore, several reports have exhibited that deletion of the Dicer enzyme or the DGCR8 gene required for miRNA biosynthesis in the skin of newborn mice might result in defective skin barrier function and epidermal basal cell hyperproliferation, implying the key role of miRNAs in epidermal development and functional maintenance [27, 28]. In diabetic-related various complications, numerous disrupted miRNAs have been validated to strongly partake in the regulation of diverse processes [29]. For example, the upregulation of miR-31-5p might improve endothelial cell function via accelerating diabetic wound healing and boosting angiogenesis [30]. Apart from that, miR-497 and miR-217 were previously validated to contribute to wound healing via decreasing inflammatory activity in diabetic animal models [31, 32]. Of interest, it has been verified that miR-204-3p might exert a protective role in HG-triggered podocyte apoptosis and dysfunction via reducing Bdkrb2 expression [33]. Beyond that, miR-204-3p has been documented to be decreased in DFU, and its aberrant expression might be correlated with the poor wound healing of this disease [34]. However, it remains unclear whether miR-204-3p modulates HG-induced endothelial cell injury in DFU. As a serine/threonine kinase, homeodomain-interacting protein kinase 2 (HIPK2) is highly expressed in HG-treated endothelial cells and boosted diabetic wound healing [35]. Here, the current work revealed that miR-204-3p possessed some potential binding sites with HIPK2. Thus, it is reasonable to assume that miR-204-3p might be involved in HG-mediated endothelial cell dysfunction via targeting HIPK2 in the current work.

Currently, several studies have indicated that nuclear factor I/C (NFIC) is the nuclear factor I family that might be implicated in the regulation of diabetic-related diseases, such as diabetic retinopathy and diabetic nephropathy [36, 37]. In terms of molecular mechanisms, NFIC might transcriptional control the target genes via binding to an *N*-terminal conserved DNA-binding domain, thereby affecting cell growth and differentiation [38–40]. Indeed, there was some early literature presenting that NFIC has a strong ability to modulate a wide range of non-coding RNAs in cell behavioral phenotypes [41, 42]. It has been reported that miRNA expression might be controlled through NFIC (a transcriptional factor) via the promoter of miRNAs [42]. Herein, the preliminary analysis of TransmiR v2.0 database discovered the putative binding sequence between NFIC and miR-204-3p. Furthermore, our data also confirmed that NFIC content was increased in ADSCs-derived exosomes. Therefore, this subject aimed to expound whether the therapeutic effects of exosomes secreted by ADSCs NFIC knockdown on HG-triggered endothelial cell dysfunction were mediated by the miR-204-3p/HIPK2 axis.

## Materials and methods

### Clinical samples and cell culture

After obtaining informed consent from all participants, skin samples were collected from 27 DFU sufferers and 27 emergency foot trauma patients without diabetes) at Yichang Central People's Hospital, the First College of Clinical Medical Science, China Three Gorges University. Meanwhile, this project was implemented after approval from the Ethics Committee of Yichang Central People's Hospital, the First College of Clinical Medical Science, China Three Gorges University.

Under the moist atmosphere with 5% CO_2_ at 37 °C, human umbilical vein endothelial cells (HUVECs, Sciencell, Carlsbad, CA, USA, cat no. #8000) were grown in specific endothelial culture medium (Sciencell, cat no. #1001). Subsequently, HUVECs were randomly divided into the normal glucose group (NG, 5 mM, Sigma-Aldrich, St. Louis, MO, USA, cat no. 50-99-7) and the high glucose group (HG, 20 mM, Sigma-Aldrich, cat no. D0822) for 72 h.

### Real-time quantitative polymerase chain reaction (RT-qPCR)

For total RNA extraction from tissue samples and HUVECs, Trizol reagent (Invitrogen, Paisley Scotland, UK, cat no. 12-183-555) was applied in this experiment. After synthesizing template DNA using miRNA First Strand cDNA Synthesis (Sangon, Shanghai, China, cat no. B532453-0020) and PrimeScript RT reagent Kit (Takara, Tokyo, Japan, cat no. RR047B), amplification reaction was carried out on a 7500 Real-time PCR system (Applied Biosystems, Foster City, CA, USA) with SYBR Green PCR Kit (Applied Biosystems, cat no. 4309155). The results were calculated by the 2^−ΔΔCt^ method and normalized to the expression level of U6 and GAPDH. Sequences were displayed in Table 1.

**Table 1:** Primers sequences used for PCR

Name		Primers for PCR (5′–3′)
NFIC	Forward	ATGTATTCGTCCCCGCTCTG
	Reverse	GTTGAACCAGGTGTAGGCGA
HIPK2	Forward	CCCCGTGTACGAAGGTATGG
	Reverse	GGGATGTTCTTGCTCTGGCT
miR-204-3p	Forward	CGATCTGCTGGGAAGGCAAAG
	Reverse	CTGGTGTCGTGGAGTCGG
U6	Forward	CTCGCTTCGGCAGCACATA
	Reverse	CGAATTTGCGTGTCATCCT
GAPDH	Forward	CAAATTCCATGGCACCGTCA
	Reverse	GACTCCACGACGTACTCAGC

### Cell transfection

In short, miR-204-3p mimic/inhibitor (miR-204-3p/In-miR-204-3p), NFIC small interfering RNA si-NFIC: 5′-AUUUUCCACCGAAAACGUGGG-3′ (sense), 5′-CACGUUUUCGGUGGAAAAUUA-3′ (antisense), and their controls (miR-NC/In-miR-NC, si-NC) from RiboBio (Guangzhou, China) were transfected into cells. The coding sequences for the mRNAs of HIPK2 (NM_022740.5) or NFIC (NM_001245002.2) were inserted into pcDNA vector (Invitrogen) to overexpress HIPK2 or NFIC in HUVECs or ADSCs. For cell transfection, Lipofectamine 3000 (Invitrogen, cat no. L3000015) was utilized for 48 h.

### Cell proliferation ability

For the cell counting kit-8 (CCK-8) assay, after various treatments, HUVECs at the logarithmic growth phase were digested and incubated for 24 h. After replacing with fresh medium, cells were mixed with 10 μL CCK-8 solution (Sigma-Aldrich, cat no. 99247-33-3) at different time points. After being cultured for 4 h, cell viability was analyzed via reading absorbance at 450 nm based on a microplate reader. For 5-ethynyl-2′-deoxyuridine (EdU) assay, 4 × 10^4^ HUVECs in 24-well plates were cultured in EdU working solution (RiboBio, cat no. C10310-1). 2 h later, PBS solution containing 4% paraformaldehyde was applied to fix cells, which then reacted with 0.5% Triton X-100. After being stained with Apollo reaction cocktail and DAPI, the cells were observed according to a fluorescence microscope.

### Cell apoptosis assay

In brief, harvested HUVECs in 6-well plates were washed with PBS at room temperature. After being subjected to the fixation with 70% ethanol on ice for 1 h, the cells suspension in binding buffer were orderly stained with 5 μL Annexin V-FITC and 10 μL PI solution (Solarbio, Beijing, China, cat no. CA1020) for 10 min in the dark. Finally, a flow cytometer was employed to identify the apoptosis cells.

### Wound healing assay

At first, the HUVECs monolayer was formed through culturing about 1 × 10^5^ cells overnight. Subsequently, a wound-mimicking straight line was made on a monolayer based on a sterile pipette tip (time 0 h). After replacement with serum-free medium, cells were continued for 24 h. At last, the gap size was observed and captured via microscopy for comparison of cell migration rates.

### Tube formation assay

The angiogenetic ability of HUVECs was assessed in this assay. Firstly, dissolved matrigel (BD Biosciences, Heidelberg, Germany, cat no. 356235) in 96-well plates were incubated for 30 min. HUVECs from different treatments were serum-starved and introduced into matrigel-coated plates in the medium for 12 h. Then, a light microscope was applied for the observation of the tube structures formed.

### Dual-luciferase reporter assay

First of all, TargetScan (https://www.targetscan.org) tool was used to generate the possible binding sites between miR-204-3p and HIPK2 3′ untranslated region (3′UTR). Then, these sequences were inserted into pmirGLO (Promega, Madison, WI, USA), generating HIPK2 3′UTR_WT_ construct_._ In parallel, HIPK2 3′UTR_MUT_ construct was acquired according to a QuikChange II site-directed Mutagenesis kit (Agilent Technologies, Santa Clara, CA, USA, cat no. 200519). These constructs were co-transfected into HUVECs along with miR-204-3p or miR-NC. For evaluating miR-204-3p promoter activity, the promoter (− 2000 to − 1 bp) immediately upstream of the transcription start site (TSS) of miR-204-3p possessing WT or MUT NFIC binding sites was introduced into pGL4-basic vector (Promega) for 48 h. Then, these vectors were co-transfected into HUVECs with pcDNA-NFIC or pcDNA-NC. Finally, HUVECs were harvested after 48 h, cell lysates were collected for the detection of luciferase activities based on Dual Luciferase Assay Kit (Promega, cat no. E1910).

### Western blot assay

Generally, the lysis of tissues, cell lines, and exosomes was prepared according to RIPA buffer (Beyotime, Shanghai, China, cat no. R0278), followed by the measurement of total protein level using a BCA kit (Takara, cat no. T9300A). After separation via 10% SDS-PAGE, the protein was shifted to PVDF membranes (Millipore, Molsheim, France), which then underwent overnight incubation at 4 °C with primary antibodies (Abcam, Cambridge, MA, USA): HIPK2 (ab108543, 1:2000), NFIC (ab245597, 1:2000), CD81 (ab109201, 1:1000), CD63 (ab134045, 1:1000), and β-Actin (ab8227, 1:1000). After being added with secondary antibody, immunoreactive proteins were visualized referring to ECL (Promega).

### Exosome detection

At first, ADSCs were prepared from human adipose tissues and characterized as previously described [19], and incubated with DMEM medium and 10% FBS. For exosome isolation, ADSCs at 80–90% confluence were cultured in EGM-2MV media for 24 h. After being removed dead cells and debris via centrifuged at 2000*g* for 10 min, the supernatant was collected and mixed with ExoQuick Exosome Precipitation Solution (System Biosciences, Palo Alto, CA, USA, cat no. NC9023885) overnight at 4 °C. After centrifugation at 1600*g* for 25 min, a pellet containing exosomes was generated and resuspended in nuclease-free water. At length, transmission electron microscopy (TEM, Hitachi, Tokyo, Japan) was applied to identify the collected exosomes, whose size was determined using nanoparticle tracking analysis (NTA). At last, these acquired exosomes were stored at − 80 °C for the subsequent assays.

### Co-culture of ADSCs and HUVECs

Transwell system with 0.4-μm pores membrane (BD Biosciences) was used for the co-culture of HUVECs and ADSCs. In short, ADSCs were placed into the upper chamber, while HUVECs were introduced and grown in the lower upper at an appropriate density. Prior to co-culture, ADSCs were pre-mixed with GW4869 (Sigma-Aldrich, cat no. 567715) for 8 h to decrease exosome release. Finally, HUVECs were co-cultured with PBS or treated ADSCs under normal or HG conditions.

### Statistical analysis

Data comparison was processed using Student’s *t*-test or one-way analysis of variance (ANOVA) with Tukey’s tests. Pearson correlation analysis was employed to assess the expression association. *P* value < 0.05 was the threshold of significance. Results were analyzed using GraphPad Prism7 software and displayed as mean ± standard deviation (SD).

## Results

### Upregulation of miR-204-3p might relieve HG-induced HUVEC injury

**Fig. 1:**
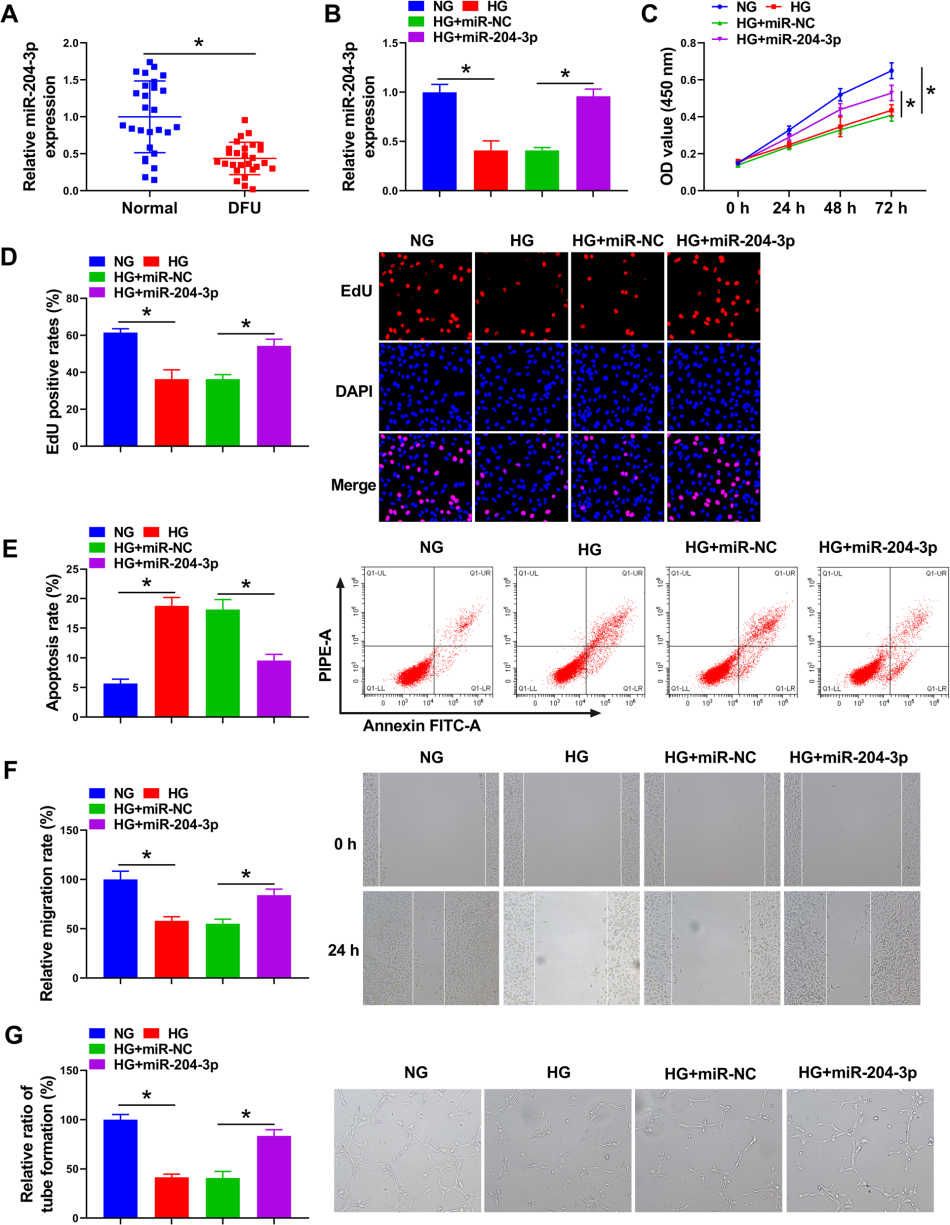
Expression patterns of miR-204-3p in DFU patients and HG-induced HUVECs. **A** miR-204-3p expression level was detected using RT-qPCR assay in 27 pairs of DFU tissues and normal tissues. **B**–**G** HUVECs were treated with NG, HG, HG + miR-NC, and HG + miR-204-3p. **B** RT-qPCR analysis of miR-204-3p expression in treated HUVECs. **C** and **D** CCK-8 and EdU assays were performed to assess HUVEC proliferative ability. **E** Flow cytometry assay was conducted to measure HUVEC apoptosis rate. **F** Wound healing assay was applied to detect HUVEC migration rate. **G** Tube formation assay was carried out to analyze HUVEC tube formation ability. *P < 0.05

At first, to investigate the function of miR-204-3p in DFU, its expression was examined using RT-qPCR assay. Results exhibited lower miR-204-3p level in 27 DFU patients than in those 27 healthy volunteers (Fig. 1A). Next, to further understand whether miR-204-3p expression in HUVECs is altered in response to diabetic stimuli, cells were exposed to hyperglycemic conditions (30 mM d-glucose). As displayed in Fig. 1B, miR-204-3p level was apparently reduced after exposure to a high-glucose environment, which was partially counteracted by miR-204-3p mimic introduction in HUVECs. Functionally, HG treatment might obviously decrease HUVEC proliferative ability, while these impacts were abolished through miR-204-3p upregulation (Fig. 1C, D). Moreover, flow cytometry assay presented that HG-induced HUVEC apoptosis rate was effectively attenuated via miR-204-3p overexpression (Fig. 1E). In parallel, wound healing results showed that elevated miR-204-3p might significantly ameliorate the repression of HG treatment on the HUVEC migration rate (Fig. 1F). In terms of angiogenesis, our data exhibited that the tube formation rate of HUVEC was remarkably blocked in response to HG treatment, which was overturned by miR-204-3p upregulation (Fig. 1G). Together, these data indicated that HG-stimulated HUVEC proliferation, migration, and angiogenesis repression were partly reversed by regulating miR-204-3p.

### miR-204-3p acted as a potential upstream regulator of HIPK2 expression

**Fig. 2:**
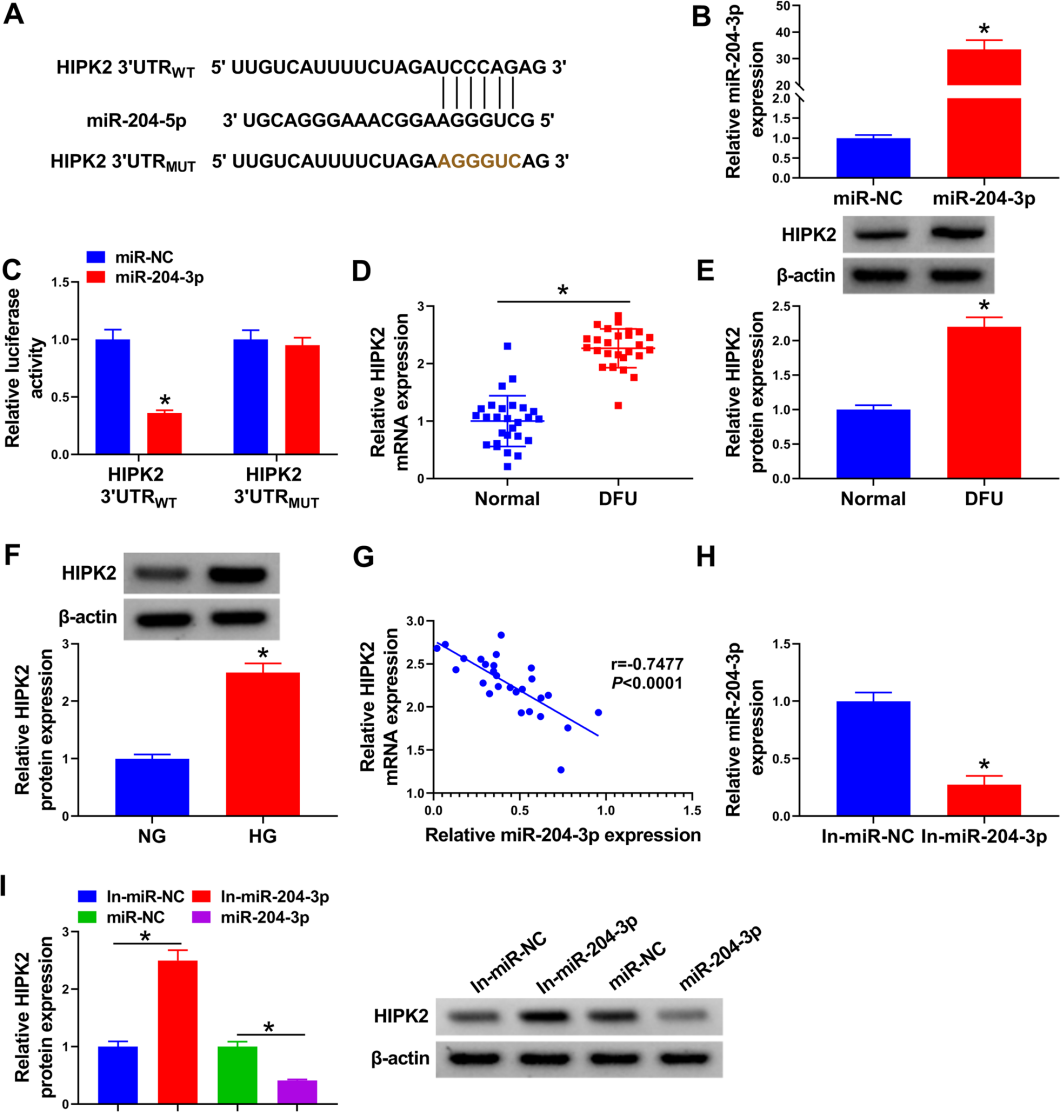
HIPK2 is the target gene of miR-204-3p. **A** Predicated results by TargetScan. **B** miR-204-3p expression was measured using RT-qPCR in HUVECs transfected with miR-204-3p or miR-NC. **C** The binding miR-204-3p to HIPK2 in HUVECs was validated by dual-luciferase reporter assay. **D** RT-qPCR analysis of HIPK2 expression in 27 DFU tissues and 27 normal tissues. **E** HIPK2 protein level was assessed using western blot assay in DFU tissues and normal tissues. **F** HIPK2 level was examined using RT-qPCR in HUVECs treated with NG or HG. **G** Pearson correlation analysis was utilized to evaluate the expression association between miR-204-3p and HIPK2 in DFU tissues. **H** Knockdown efficiency of miR-204-3p in HUVECs was assessed using RT-qPCR. **I** HIPK2 protein level was determined in HUVECs transfected with In-miR-NC, In-miR-204-3p, miR-NC, or miR-204-3p using western blot assay. *P < 0.05

Furthermore, TargetScan was applied to predicate the candidate-binding mRNAs of miR-204-3p. As a result, there were some binding sites between miR-204-3p and HIPK2 (Fig. 2A). Meanwhile, the overexpression efficiency of miR-204-3p in HUVECs was measured and exhibited in Fig. 2B. After that, a dual-luciferase reporter assay displayed that elevated miR-204-3p might prominently impede the luciferase activity in HIPK2 3′UTR_WT_, rather than the mutant group in HUVECs (Fig. 2C). Interestingly, we found that the mRNA level and protein level of HIPK2 is clearly enhanced in DFU patients relative to the normal group (Fig. 2D, E). Consistently, higher HIPK2 expression was verified in the HG group than that in NG group (Fig. 2F). Notably, our data presented that miR-204-3p level was negatively associated with HIPK2 in DFU subjects (Fig. 2G). In addition, RT-qPCR results showed that miR-204-3p content was significantly decreased in in-miR-204-3p-transfected HUVECs (Fig. 2H), indicating that the knockdown efficiency was available. Beyond that, western blot assay displayed that the HIPK2 protein level was greatly improved by miR-204-3p knockdown and evidently declined via miR-204-3p overexpression in HUVECs (Fig. 2I). Collectively, these data implied that miR-204-3p might bind to HIPK2 to hinder its expression in HUVECs.

### miR-204-3p/HIPK2 regulated HG-mediated HUVEC behaviors

**Fig. 3:**
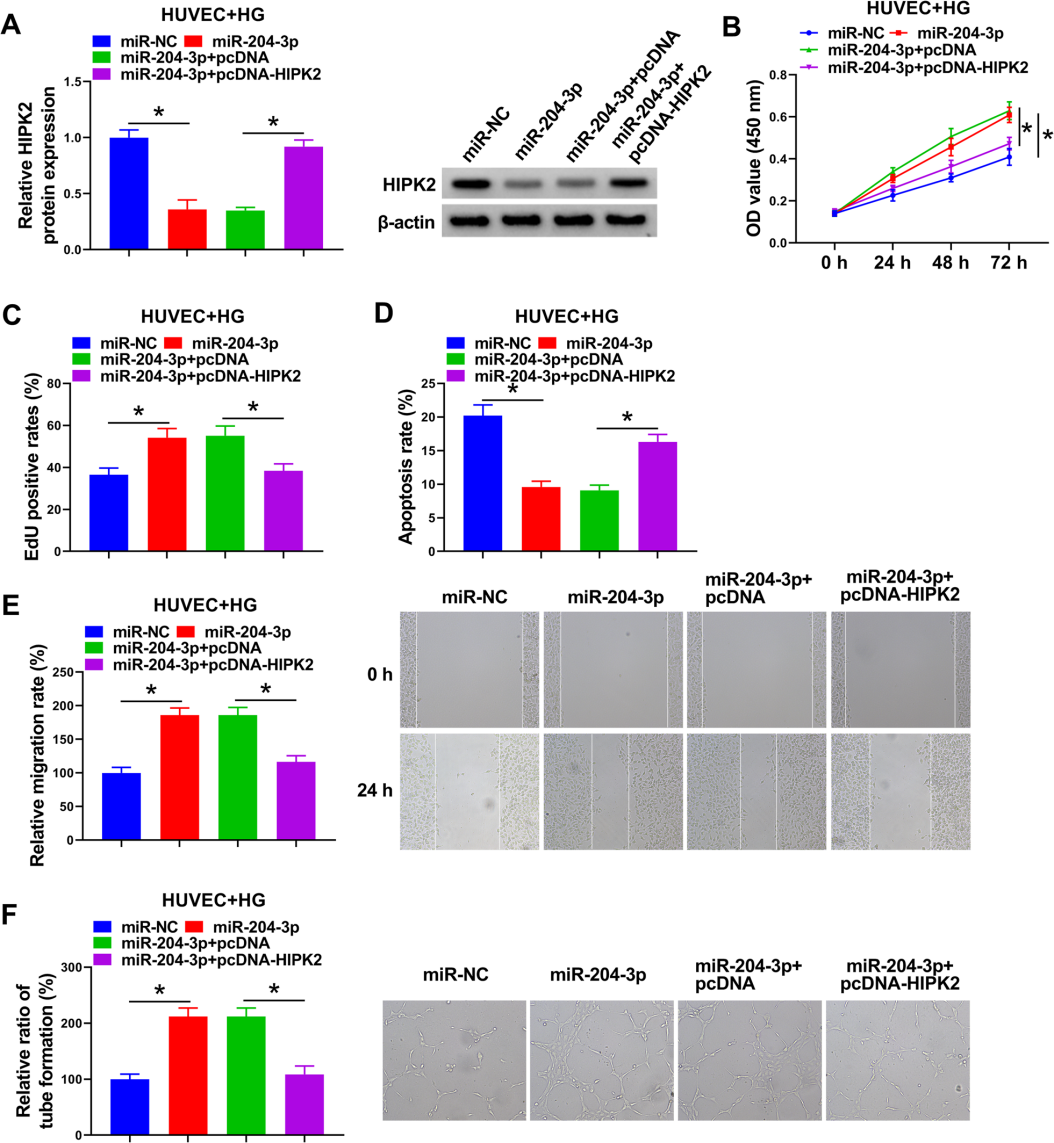
HIPK2 overexpression might abolish the influences of miR-204-3p on HG-mediated HUVEC behaviors. HUVECs were transfected with miR-NC, miR-204-3p, miR-204-3p + pcDNA, and miR-204-3p + pcDNA-HIPK2, followed by HG treatment. **A** Western blot analysis of HIPK2 protein level in treated HUVECs. **B** and **C** HUVEC proliferative ability was analyzed using CCK-8 and EdU assays. **D** HUVEC apoptosis rate was examined using flow cytometry assay. **E** HUVEC migration rate was assessed using wound healing assay. **F** HUVEC tube formation ability was evaluated using Tube formation assay. *P < 0.05

Considering the regulatory role of miR-204-3p in HIPK2 expression in HUVECs, we further explored whether the impacts of miR-204-3p on HG-triggered cell behaviors were correlative with HIPK2. As shown in Fig. 3A, the introduction of pcDNA-HIPK2 might remarkably counteract the suppression of miR-204-3p on HIPK2 protein level in HUVECs under HG conditions. Functional analysis discovered that miR-204-3p upregulation-mediated cell proliferation promotion (Fig. 3B, C) and apoptosis rate inhibition (Fig. 3D) in HG-treated HUVECs were significantly ameliorated by HIPK2 overexpression. In parallel, enhanced miR-204-3p might boost the migration rate in HG-exposed HUVECs, while this protection was partially overturned through HIPK2 upregulation (Fig. 3E). Apart from that, pcDNA-HIPK2 introduction also abrogated the promotion of miR-204-3p on angiogenesis in HG-treated HUVECs (Fig. 3F). In total, these data suggested that miR-204-3p might facilitate proliferation, migration, and angiogenesis in HG-induced HUVECs by regulating HIPK2.

### miR-204-3p expression was transcriptionally activated by NFIC

**Fig. 4:**
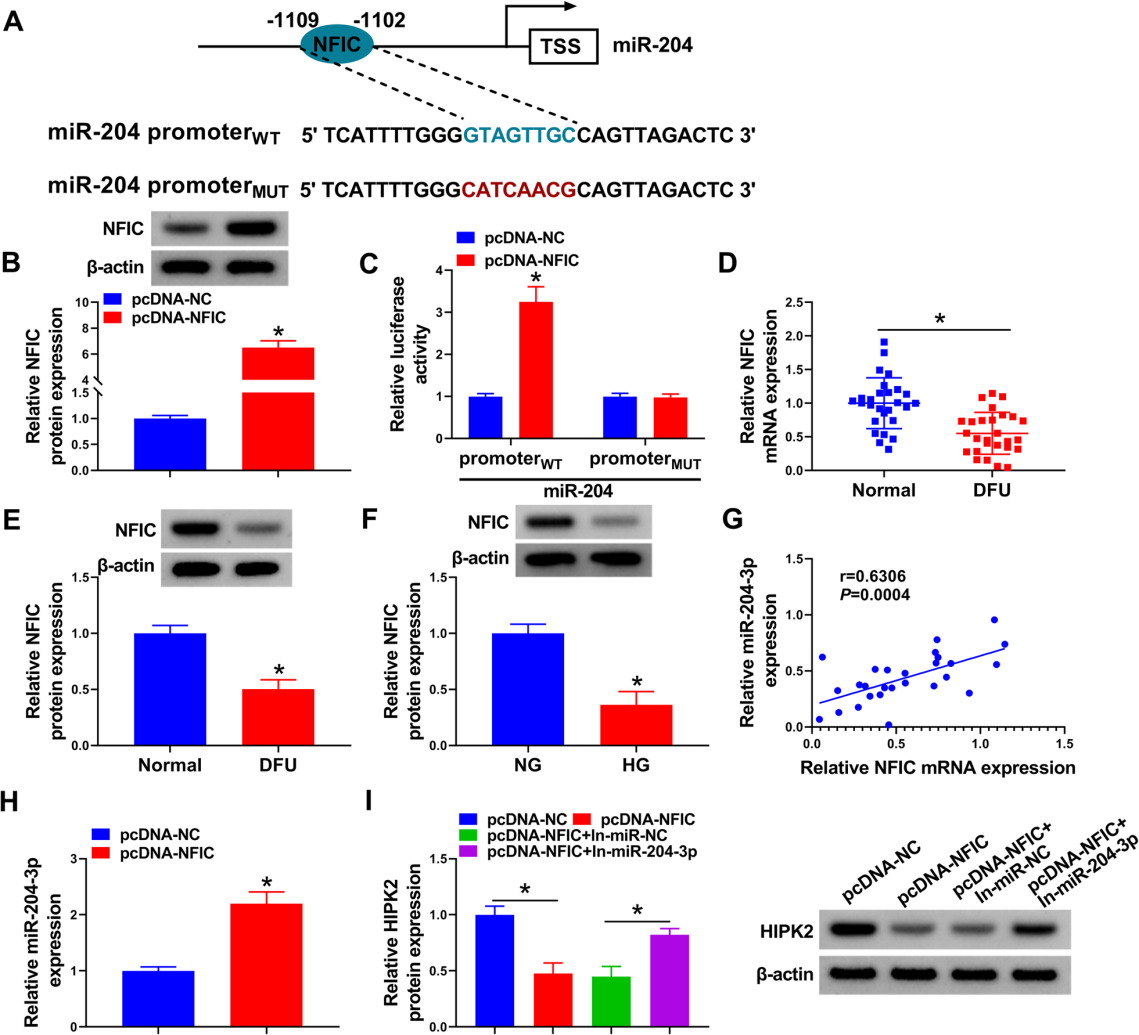
NFIC increased miR-204-3p expression. **A** Binding sites and mutation sites of transcription factor NFIC and miR-204 promoter sequence are shown. **B** Overexpression efficiency of NFIC was detected using western blot assay. **C** A dual-luciferase reporter assay was applied to analyze the binding between NFIC and miR-204 promoter. **D** and **E** RT-qPCR and western blot assays were employed to measure the expression level of NFIC in DFU tissues and normal tissues. **F** Western blot analysis of NFIC protein level in HUVECs treated with HG or NG. **G** Expression correlation between miR-204 and NFIC in DFU patients was analyzed using Pearson correlation analysis. **H** Effect of NFIC upregulation on miR-204 expression in HUVECs was monitored by RT-qPCR. **I** Western blot analysis of HIPK2 protein level in HUVECs transfected with pcDNA-NC, pcDNA-NFIC, pcDNA-NFIC + In-miR-NC, and pcDNA-NFIC + In-miR-204-3p. *P < 0.05

Previous studies have indicated that NFIC, a regulatory transcription factor, has a site-specific DNA-binding function in the regulation of gene expression [43]. It has been reported that NFIC might recognize the promoter of some miRNAs, which improved the transcription of miRNAs [42]. Here, TransmiR v2.0 website database was applied to preliminarily predict NFIC might act as a potential transcription factor of miR-204-3p (Fig. 4A). Besides, western blot results exhibited that the introduction of pcDNA-NFIC might obviously enhance the protein level of NFIC in HUVECs (Fig. 4B), suggesting the overexpression efficiency is successful. In order to check whether NFIC might directly bind to the miR-204-3p promoter, a dual-luciferase reporter assay was performed in HUVECs. Firstly, we sub-cloned the WT and MUT of the miR-204-3p promoter possessing − 1114 to − 1096 relative to TSS, which contained a binding site. As shown in Fig. 4C, the promoter activity of miR-204-3p was greatly improved in WT after NFIC overexpression, rather than MUT-transfected HUVECs. Furthermore, NFIC content was markedly decreased in DFU patients compared with the normal group (Fig. 4D, E). Similarly, the remarkable downregulation of NFIC was observed in the HG group versus the NG group (Fig. 4F). Additionally, our data verified that NFIC level was positively correlated with miR-204-3p in NFIC subjects (Fig. 4G). Synchronously, RT-qPCR assay presented that the upregulation of NFIC might strikingly strengthen miR-204-3p level in HUVECs (Fig. 4H). Besides, western blot results showed that miR-204-3p knockdown might effectively abolish the repression of pcDNA-NFIC on HIPK2 protein level in HUVECs (Fig. 4I), implying that NFIC might regulate HIPK2 expression via interacting with miR-204-3p. Overall, these findings discovered that NFIC positively regulates miR-204-3p expression via binding to its promoter region.

### Exosomes from ADSCs overexpressing NFIC might improve miR-204-3p expression in HUEVCs

**Fig. 5:**
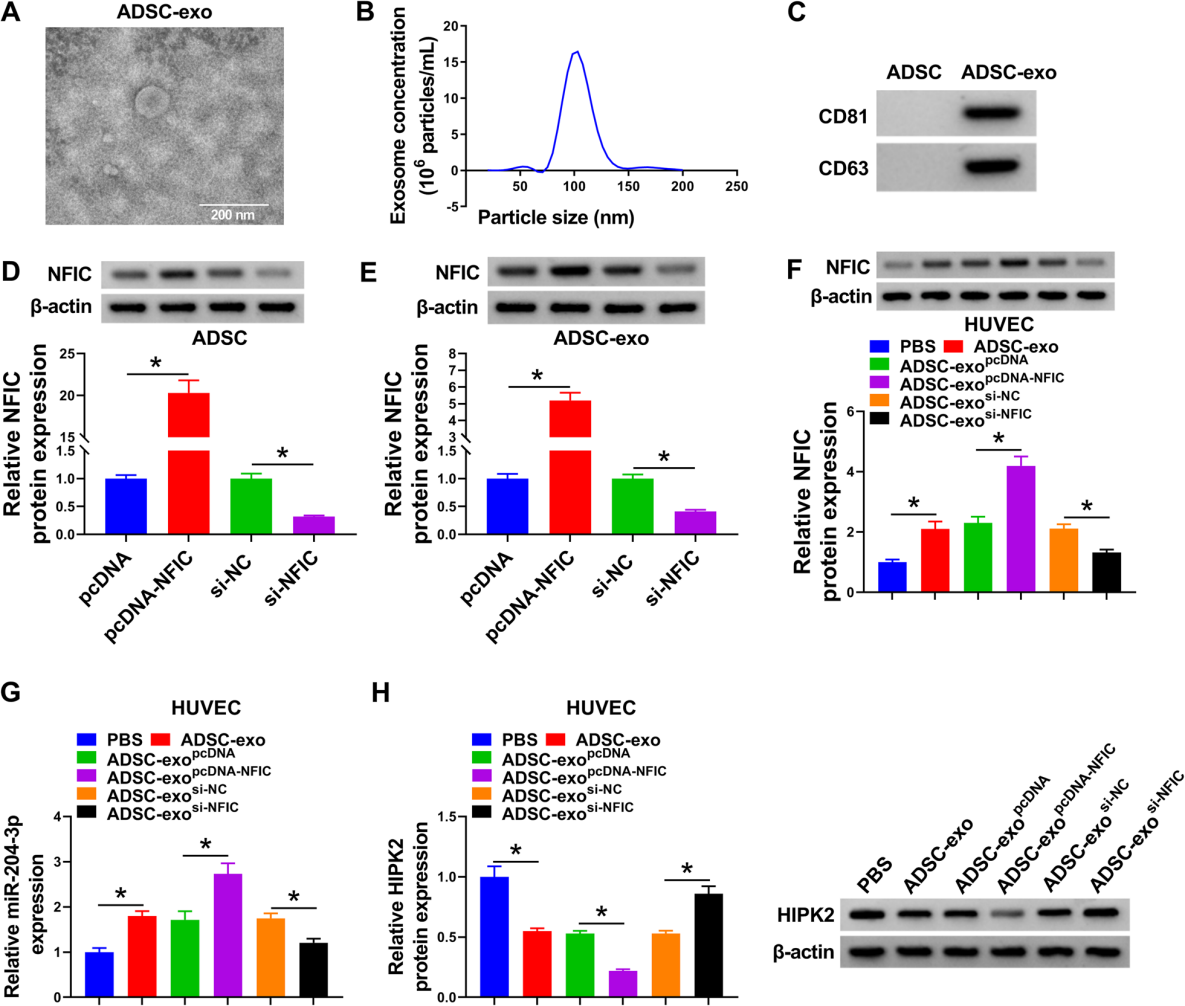
Transfer of ADSC exosome NFIC to HUEVC promotes the expression of miR-204-3p. **A** and **B** The representative micrograph of round-shaped vesicles by TEM. **B** Concentration and size distribution of exosomes was examined using NTA. **C** CD81 and CD63 protein levels in ADSC and ADSC-exo were assessed using western blot assay. **D** Overexpression or knockdown efficiency of NFIC in ADSCs was determined by western blot assay. **E** NFIC protein level was measured in ADSC-exo with pcDNA, pcDNA-NFIC, si-NC, or si-NFIC using western blot assay. (F–H) HUVECs were incubated with PBS, ADSC-exo, ADSC-exo^pcDNA^, ADSC-exo^pcDNA−NFIC^, ADSC-exo^si−NC^, or ADSC-exo^si−NFIC^. **F** Western blot analysis of NFIC protein level in HUVECs. **G** miR-204-3p expression was detected using RT-qPCR in HUVECs. **H** HIPK2 protein level was measured using western blot assay in HUVECs. *P < 0.05

Furthermore, to investigate the mediating roles of exosomes in ADSCs-triggered DFU progression repression, the exosomes from cultured human ADSCs were isolated and identified. As shown in Fig. 5A and B, these exosomes possess round or oval membranes under the TEM, and the diameters of most particles were within the range of 50–200 nm using NTA analysis. Furthermore, western blot assay presented significant expression of exosome biomarkers CD81 and CD63 in ADSCs-derived exosomes, relative to the ADSCs group (Fig. 5C). Subsequently, in order to evaluate the anti-DFU roles of ADSC-derived exosomes and NFIC in these exosomes, NFIC was knocked down or overexpressed in these ADSCs. The transfection efficiency was detected and illustrated in Fig. 5D. Moreover, western blot results exhibited that NFIC expression in exosomes secreted by the ADSCs transfected with pcDNA-NFIC was obviously enhanced relative to that in the pcDNA group, conversely, NFIC level was apparently reduced in exosomes from ADSCs with si-NFIC compared with the si-NC group (Fig. 5E). Later on, to further check the influence of ADSC-exo^pcDNA−NFIC^ or ADSC-exo^si−NFIC^ on HUVECs, HUVECs were co-cultured with these transfected exosomes or PBS. Data showed that NFIC and miR-204-3p levels in HUVECs were clearly improved in the ADSC-exo^pcDNA−NFIC^ group and evidently reduced in the ADSC-exo^si−NFIC^ group compared with their corresponding control groups (Fig. 5F and G). However, HIPK2 protein level presented the opposite trend in HUVECs (Fig. 5H). In summary, these data suggested that exosomes from NFIC-overexpressing ADSCs increased miR-204-3p level in HUEVCs.

### Exosomes from NFIC-silencing ADSCs facilitated HG-mediated HUVEC proliferation, migration, and angiogenesis by regulating miR-204-3p

**Fig. 6:**
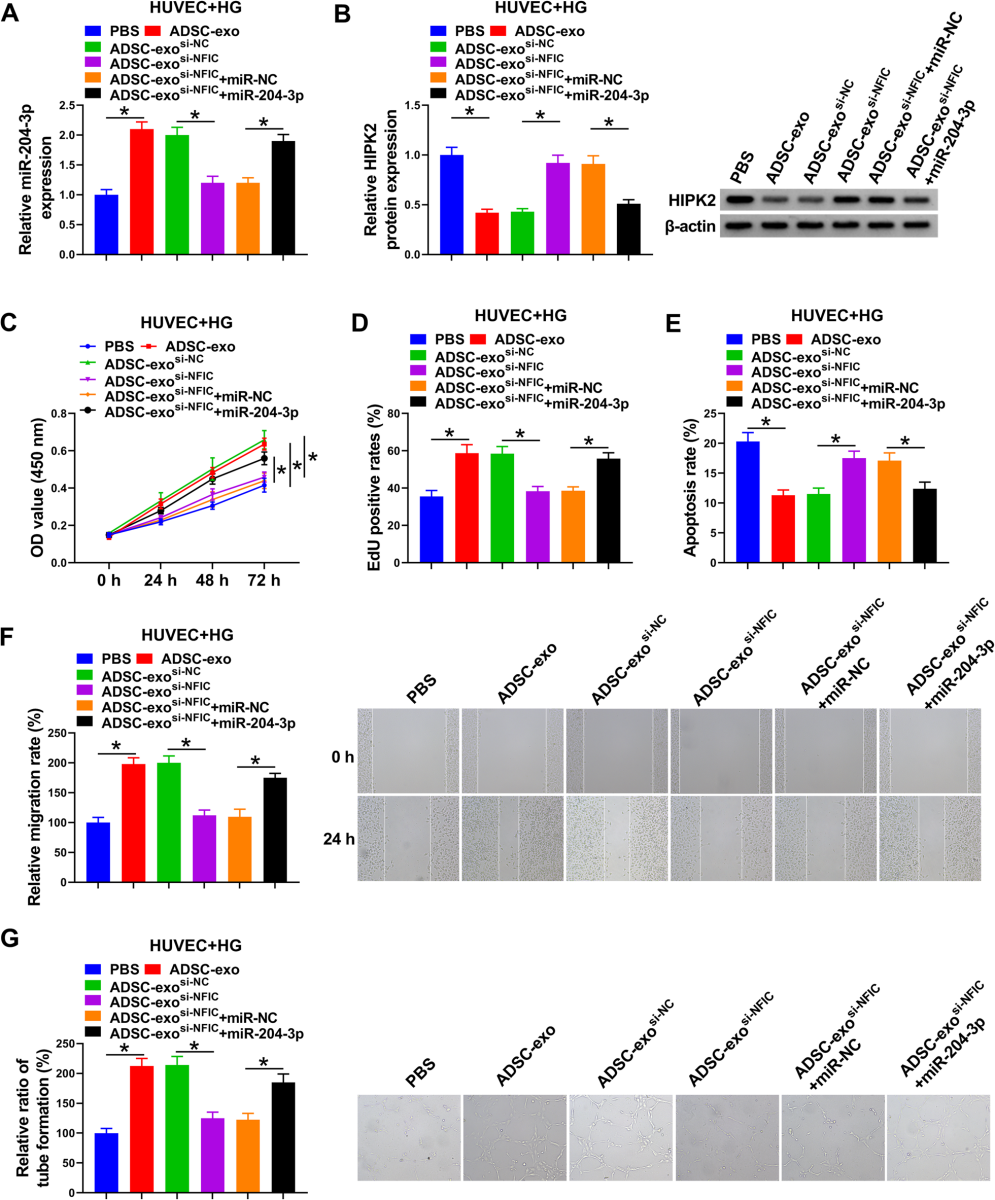
ADSC-exo si-NFIC expedited HG-mediated HUVEC proliferation, migration, and angiogenesis by regulating miR-204-3p. HUVECs were incubated with PBS, ADSC-exo, ADSC-exo^si−NC^, ADSC-exo^si−NFIC^, ADSC-exo^si−NFIC^ + miR-NC, or ADSC-exo^si−NFIC^ + miR-204-3p, followed by HG treatment. **A** RT-qPCR analysis of miR-204-3p expression in treated HUVECs. **B** Western blot analysis of HIPK2 protein level in HUVECs. **C** and **D** CCK-8 and EdU analysis of HUVEC proliferative ability. **E** Flow cytometry analysis of HUVEC apoptosis rate. **F** Wound healing analysis of HUVEC migration rate. **G** Tube formation analysis of HUVEC tube formation ability. *P < 0.05

Subsequently, we further explored whether the impacts of ADSC-exo^si−NFIC^ on HG-HUVEC behaviors were mediated by miR-204-3p/HIPK2. First of all, RT-qPCR assay showed that the introduction of miR-204-3p might partially abrogate the inhibition of ADSC-exo^si−NFIC^ on miR-204-3p level in HG-treated HUVECs (Fig. 6A). In parallel, ADSC-exo^si−NFIC^ might distinctly reinforce the protein level of HIPK2 in HG-exposed HUVECs, which was reversed by miR-204-3p upregulation (Fig. 6B). Functionally, decreased cell proliferation (Fig. 6C, D) and elevated cell apoptosis rate (Fig. 6E) due to ADSC-exo^si−NFIC^ in HG-mediated HUVECs were significantly ameliorated via miR-204-3p overexpression. Apart from that, wound healing assay verified that ADSC-exo^si−NFIC^ might apparently repress cell migration ability in HG-treated HUVECs, whereas this phenomenon was relieved after the introduction of miR-204-3p (Fig. 6F). Meanwhile, the suppression of ADSC-exo^si−NFIC^ on HUVEC tube formation ability under HG conditions was partly mitigated via miR-204-3p overexpression (Fig. 6G). All of these results illuminated that the regulatory role of exosomes from NFIC-deficiency ADSCs on HG-caused HUVEC proliferation, migration, and angiogenesis was regulated by miR-204-3p.

## Discussion

As one of the most common serious diabetic complications, DFU has been responsible for the majority of leg amputations in diabetic individuals [44]. At present, the common etiopathogenesis of DFU is ascribed to the dysfunctions of the neural systems and the impairment of blood vessels [3, 45]. Nowadays, some reports have shown that the failure of wound healing in DFU sufferers is closely associated with abnormal alterations in a variety of biological processes, particularly impaired angiogenesis [46, 47]. Nevertheless, the underlying molecular pathogenic mechanism remains elusive. The current research fundamentally demonstrated a new mechanism by which NFIC-exosome loaded into the ADSC sheet that might promote miR-204-3p expression to repress HIPK2 level and modulate HG-induced HUVEC proliferation, migration, and angiogenesis, thereby affecting DFU healing.

Here, our data verified that a wound healing-related miRNA, miR-204-3p, was poorly expressed in DFU patients. It has been widely accepted that DFU is more difficult to heal than non-diabetic chronic skin wounds, and that hyperglycemia is a significant adverse factor. Hence, in conjunction with our data, it is reasonable to assume that hyperglycemia might have a detrimental impact on DFU via altering miR-204-3p expression. Furthermore, earlier literature displayed that miR-204-3p might exert a protective role in HG-triggered keratinocyte apoptosis and dysfunction via reducing KLF6 content [34]. Angiogenesis is essential for promoting wound repair via allowing oxygen and nutrients to arrive at the wound sites [48]. Endothelial cell dysfunction, a critical driver of damaged DFU healing, is strongly related to impaired angiogenesis [49, 50]. In this paper, our data identified that miR-204-3p content was decreased in HUVECs under HG environment, and its upregulation might partially weaken HG-aroused HUVEC proliferation, migration, and angiogenesis repression in vitro. In general, miRNAs are capable of modulating physiological and pathological processes via repressing target mRNAs [51]. Here, based on bioinformatics prediction and a series of experiments, HIPK2 was deemed as a downstream target of miR-204-3p. Recent studies unveiled that HG condition played a vital influencing factor in modulating HIPK2 content. For instance, it is found that HIPK2 level was improved in HG-treated mouse glomerular mesangial cells in a diabetic nephropathy research [52]. Apart from that, HIPK2 silencing might overturn HG-hindered HUVEC migration and angiogenesis [35]. Consistent with these reports, our data displayed that HIPK2 content was clearly enhanced in DFU subjects and HG-mediated HUVECs. Functionally, elevated HIPK2 might abolish the repression of miR-204-3p on HG-triggered HUVEC dysfunction in vitro. From the above findings, it is concluded that miR-204-3p has a protective effect on HG-induced HUVEC functional impair via directly targeting HIPK2.

Despite the abnormal regulation of miR-204-3p implicated in HG-mediated HUVEC damage, definitive evidence for an explanation of this dysregulation is lacking. Interestingly, work in many laboratories has widely investigated that the transcriptional modulation of non-coding RNAs is mediated by several transcription factors, including NFIC [41, 42]. Herein, miR-204-3p as a target of NFIC was uncovered according to bioinformatics analysis. NFIC specifically bound to the promoter region of miR-204-3p and increased its expression in HUVECs. Furthermore, it has been reported that overexpressing NFIC might relieve HG-triggered inflammation and fibrosis in mouse podocytes in diabetic nephropathy [37]. Of note, ADSCs-derived exosomes have been validated to possess wound healing via accelerating HUVEC proliferation and angiogenesis [53]. Synchronously, exosomes secreted by ADSCs might enhance endothelial cell angiopoietins under HG environment, thereby expediting diabetic wound healing [19, 54]. In this paper, our data suggested that exosomes from ADSCs NFIC-upregulating might improve the expression of miR-204-3p and inhibit HIPK2 level in HUVECs. More importantly, functional analysis revealed that exosomes from ADSCs silencing NFIC might restrain HUVEC angiopoietins via targeting miR-204-3p/HIPK2 under HG treatment. These findings supported the potential for miR-204-3p-mediated HIPK2 repression as the mechanism underpinning the promoting role of NFIC-loaded ADSC-derived exosomes in wound healing. However, this study had some limitations. For instance, we did not perform animal experiments and more clinical trials will be needed in the future.

## Conclusion

In summary, these data outlined that exosomes from ADSC overexpressing NFIC might attenuate HG-induced HUVEC damage via regulating miR-204-3p/HIPK2, providing a fresh perspective for the development of future therapeutic approaches against DFU.

## Data Availability

The data sets used and/or analyzed during the current study are available from the corresponding author on reasonable request.
